# Residual Attention-Based Image Fusion Method with Multi-Level Feature Encoding

**DOI:** 10.3390/s25030717

**Published:** 2025-01-24

**Authors:** Hao Li, Tiantian Yang, Runxiang Wang, Cuichun Li, Shuyu Zhou, Xiqing Guo

**Affiliations:** 1 Aerospace Information Research Institute, Chinese Academy of Sciences, Beijing 100094, China; lihao231@mails.ucas.ac.cn (H.L.); licc@aircas.ac.cn (C.L.); zhousy@aircas.ac.cn (S.Z.); 2 University of Chinese Academy of Sciences, Beijing 100049, China; yangtiantian23@csu.ac.cn; 3 Technology and Engineering Center for Space Utilization, Chinese Academy of Sciences, Beijing 100094, China; 4 School of Future Technology, Tianjin University, Tianjin 300072, China; wrx_7881@tju.edu.cn

**Keywords:** image fusion, transformer, cross attention, deep learning, feature encoding

## Abstract

This paper presents a novel image fusion method designed to enhance the integration of infrared and visible images through the use of a residual attention mechanism. The primary objective is to generate a fused image that effectively combines the thermal radiation information from infrared images with the detailed texture and background information from visible images. To achieve this, we propose a multi-level feature extraction and fusion framework that encodes both shallow and deep image features. In this framework, deep features are utilized as queries, while shallow features function as keys and values within a residual cross-attention module. This architecture enables a more refined fusion process by selectively attending to and integrating relevant information from different feature levels. Additionally, we introduce a dynamic feature preservation loss function to optimize the fusion process, ensuring the retention of critical details from both source images. Experimental results demonstrate that the proposed method outperforms existing fusion techniques across various quantitative metrics and delivers superior visual quality.

## 1. Introduction

Due to the limitations of imaging technology and the finite information content of a single image, capturing both the salient features and fine details of a target in one image remains a significant challenge. However, fields such as military reconnaissance, autonomous driving, and object detection demand high-quality and comprehensive image information, sparking widespread research interest in image fusion technology [1,2,3]. Among the various fusion techniques, combining infrared (IR) and visible (VI) light images has emerged as a key research focus. Infrared images excel at highlighting the thermal characteristics of target objects, improving target detection, while visible light images provide richer textures and detailed color information. Advances in image fusion technology are crucial for further enhancing the performance of these application areas.

Early developments in image fusion date back to the 1990s, when researchers began exploring traditional fusion techniques. Johnson [4] and Broussard [5] introduced a neural model called the Pulse-Coupled Neural Network (PCNN), which was first applied to image fusion two years later. The application of PCNN in image fusion is characterized by two main approaches: applying the model exclusively to the high-frequency subbands of an image or to both high- and low-frequency subbands simultaneously. The PCNN model effectively mitigated the issue of detail loss commonly associated with traditional fusion techniques. However, its intricate network architecture and complex parameter configurations presented challenges, particularly in terms of computational efficiency and generalization ability, which required further improvement. Approximately two decades later, Liu et al. [6] pioneered the application of Convolutional Neural Networks (CNNs) to image fusion tasks. CNN-based methods overcame the limitations of traditional algorithms that required manually designed fusion rules. Nonetheless, the complexity of the loss function design in CNNs still constrains their overall performance. For instance, Zhang et al. [7] introduced the PMGI framework, which utilizes an end-to-end network to model the fusion problem as one of preserving both texture and pixel intensity. Similarly, Ma et al. [8] proposed STDFusionNet, which integrates residual modules into the network to enhance infrared target visibility against visible light backgrounds. Furthermore, Xu et al. [9] developed U2Fusion, a unified end-to-end image fusion network that employs elastic weight consolidation to significantly reduce temporal and spatial complexities during training. Generative Adversarial Networks (GANs) have also been applied to image fusion. Ma et al. [3] leveraged the GAN framework introduced by Goodfellow et al. [10] for fusing infrared and visible light images. They later improved this approach by proposing DDcGAN [11], which replaced the single generator and single discriminator architecture with a single generator and dual discriminators to address the issue of modality bias in fusion results. More recently, Zhao et al. [12] proposed DDFM, a fusion algorithm based on Denoising Diffusion Probabilistic Models (DDPMs). This redefined the image fusion process as a conditional generation problem under the DDPM sampling framework. However, GAN-based methods still face challenges, such as training instability [13,14], and determining the optimal number of training iterations for the generator and discriminator remains difficult. In addition to CNNs and GANs, Autoencoders (AEs) have also been extensively studied for image fusion. For example, DeepFuse [15], proposed by Prabhakar et al., is a notable AE-based model initially designed for multi-focus image fusion. Despite its original purpose, the model has demonstrated effectiveness in the fusion of infrared and visible light images as well.

This paper focuses on image fusion methods based on Transformers, which were originally introduced by Vaswani et al. [16] in 2017 for natural language processing (NLP) tasks. In 2020, Dosovitskiy et al. [17] adapted Transformers for computer vision applications. Their self-attention mechanisms allow Transformers to effectively capture long-range dependencies in images, which is critical for understanding complex image content. Compared to traditional Convolutional Neural Networks (CNNs), Transformers process information more efficiently in parallel, significantly accelerating both training and inference. These advantages have been demonstrated in models such as SwinFusion [18], CDDFuse [19], and DATFuse [20], which have achieved impressive results in the fusion of infrared (IR) and visible (VI) light images. Despite the remarkable performance of existing Transformer-based image fusion methods, several limitations persist. First, the attention mechanism, a core component of Transformers, primarily focuses on global information while often neglecting fine-grained local details from the input images. Second, the Transformer architecture was originally designed for NLP tasks, where sentences are tokenized into semantically meaningful units based on words. However, when adapted to image processing, images are typically divided into simple patches that serve as tokens, which do not inherently convey semantic meanings analogous to those in NLP. Third, in the field of image fusion, there is a strong demand to preserve more texture details from the original images while maintaining a high level of consistency between the fused image’s style and its input sources. However, current loss functions lack the adaptability to dynamically adjust fusion objectives based on the varying properties of input images. Finally, beyond image fusion, deep learning has also been applied to analyze sensor data for structural health monitoring of buildings and bridges. By identifying abnormal patterns, potential safety issues or structural damage can be detected early, enabling timely maintenance and repairs [21,22].

To address the aforementioned issues, this paper proposes an end-to-end image fusion model called the Residual Attention-Based Image Fusion Method with Multi-Level Feature Encoding (MFRA-Fuse). To achieve the semantically meaningful tokenization of images and better preservation of local details, we introduce a Deep Semantic Encoding (DSE) module based on RepVGG [23]. However, as Transformers inherently focus more on global information, there is a risk that the semantic space generated by DSE may deviate significantly from the original image, resulting in a loss of fine details after attention computation. To mitigate this, we propose a multi-scale semantic encoding structure, where the query utilizes deep semantic encoding, while the key and value adopt traditional shallow semantic encoding, ensuring a balance between the global context and local detail preservation. Additionally, a residual cross-attention module is introduced to retain the original image information throughout the fusion process. Finally, to better handle image fusion tasks under varying brightness and environmental conditions, we design a novel loss function. This loss function dynamically assigns pixel weights based on image gradients and ensures consistency in color style across fused images, improving the adaptability and robustness of the model.

The main contributions of this paper are summarized as follows:

- A deep–shallow semantic encoding structure is proposed to provide more appropriate semantic representations for attention computation while ensuring the preservation of the original image information during the fusion process.
- A multi-level cascaded semantic encoding structure based on RepVGG is introduced, which is specifically tailored for image fusion applications, providing semantically meaningful query encoding.
- A residual cross-attention module is designed to retain the original image details during the fusion process, preventing the attention mechanism from overlooking fine-grained information during long-range computations.
- A novel pixel-level loss function is proposed, which dynamically adjusts based on image texture and color information, enabling the network to generalize effectively across diverse fusion scenarios.

## 2. Related Work

### 2.1. Traditional Infrared and Visible Image Fusion Methods

Over the past few decades, researchers have proposed numerous traditional methods for fusing infrared and visible light images. These methods include approaches based on sparse representation (SR) [24,25], multi-scale transform (MST) [26,27], saliency detection [28,29], and subspace-based algorithms [30]. Each of these methods processes images from distinct perspectives to achieve fusion, enriching the resulting fused images with detailed textures and abundant information.

Sparse representation (SR)-based fusion methods include techniques such as joint sparse representation (JSR) [31] and convolutional sparse representation (CSR) [32]. These methods employ learned sparse dictionaries to represent the source images as sparse linear combinations of dictionary atoms, followed by weighted combinations [33] to generate the fused image. Among the traditional methods, multi-scale transform (MST)-based approaches have gained particular popularity. These include wavelet-based methods [34], pyramid-based methods [35], multi-scale geometric representation methods [36], and curvelet-based methods [37]. These approaches typically decompose the source images using a common multi-scale decomposition technique, fuse the resulting low-frequency and high-frequency components, and then apply the corresponding inverse transform to reconstruct the fused image [38]. Subspace-based methods, such as those based on principal component analysis (PCA) [39] and non-negative matrix factorization (NMF) [40], are also widely employed. These methods decompose the source images into bases and coefficients, merge the bases and coefficients within an appropriate subspace, and then reconstruct the fused image. Such techniques effectively integrate structural information and texture details from different source images, producing high-quality fusion results [40]. Moreover, researchers have explored hybrid methods [41] and optimization-based methods [42] that combine the strengths of various approaches to develop fusion strategies tailored to specific application scenarios. While traditional methods have demonstrated success in fusing image details and features from multiple perspectives, they also exhibit significant limitations. These methods require the manual design of complex feature extraction and fusion rules, relying heavily on the researchers’ expertise and experiential judgment. Additionally, manually designed features often fail to capture the high-level semantic information within images, which limits the generalization capability of the resulting models [43].

### 2.2. Infrared and Visible Image Fusion Based on Deep Learning

Owing to the formidable capabilities of deep learning in automatic feature extraction, end-to-end solutions, generalization, and computational efficiency, it has been widely applied to various computer vision tasks, including person re-identification [44,45], image super-resolution [46], and image fusion. In the realm of infrared and visible light image fusion, deep learning-based methods can be broadly categorized into four main types: CNN-based methods [47,48], GAN-based methods [49,50,51], AE-based methods [52,53,54], and Transformer-based methods [18,20]. CNN-based fusion methods represent one of the most widely used approaches. These methods employ Convolutional Neural Networks to learn image features, followed by a fusion strategy to combine multiple input images into a single fused output. By leveraging the powerful feature learning capabilities of CNNs, these methods automatically extract important features from input images, thereby achieving high-quality image fusion. Most CNN-based methods adopt an end-to-end training paradigm, in which pairs of infrared and visible light images are input into the network. The network subsequently extracts features and fuses them through a specific fusion strategy to produce the final fused image. The performance of these methods is heavily influenced by the design of loss functions, which determine the type and amount of information retained in the final fused image. Despite their successes, current CNN-based end-to-end infrared and visible light image fusion networks primarily focus on pixel-level details, often neglecting a more comprehensive consideration of deep features and fine-grained information within the images. To address this limitation, MAPFusion [55] introduces a multi-level adaptive perceptual loss, a feature-level strategy that constrains the features of both the original and fused images. This approach simultaneously preserves low-level positional data and high-level semantic information, thereby optimizing CNN-based image fusion methods and enhancing their overall performance.

Furthermore, Generative Adversarial Networks (GANs) have demonstrated the ability to generate fused images that exhibit the characteristics of real images. GAN-based methods primarily rely on the adversarial interaction between the generator and the discriminator to optimize the model. The generator is responsible for producing fused images that preserve the intensity information of infrared images and the gradient details of visible light images, while the discriminator enforces the generator to create images with enhanced texture details [3]. This adversarial process aims to generate fused images that are indistinguishable from real ones, thereby meeting the requirements of image fusion. However, the design of the discriminator’s loss function often biases the fused image towards the visible light modality, with the infrared modality’s information primarily retained through content loss. This imbalance can lead to the loss of critical infrared image information. To address this issue, DDcGAN [11] improved the GAN framework by replacing the single-generator, single-discriminator architecture with a single-generator, dual-discriminator design. This adjustment mitigates the tendency of GAN-fused images to favor one specific modality over the other. Similarly, GANMcC [49], inspired by the PMGI method, divides the input into gradient and contrast channels. Each channel’s input is a weighted combination of infrared and visible light images based on specific rules. In this framework, the discriminator functions as a classifier, outputting a probability to determine whether the image belongs to the infrared or visible light category. Through adversarial training, the final fused image achieves a better balance between the two modalities. Despite these advancements, GAN-based methods still face challenges, particularly instability during the training process [13,14]. The adversarial interaction between the generator and discriminator can lead to issues such as non-convergence, slower training speeds, and difficulty in determining appropriate training steps, making stability an ongoing challenge in GAN-based image fusion.

Autoencoder (AE)-based methods inherit the traditional image fusion process, which involves three main steps: feature extraction, feature fusion, and feature reconstruction. AE-based methods are unsupervised approaches that do not require labeled training data. Instead, they utilize large image datasets to train an encoder–decoder architecture. The encoder is responsible for extracting features from the input images, while the decoder reconstructs the fused image. During this process, the extracted features are combined using specifically designed fusion rules, and the decoder reconstructs the final fused image. Successful reconstruction of the original input image indicates that the encoder has effectively captured the essential features from the source images. Classical AE-based methods include DeepFuse, DenseFuse [56], and VAEFuse [57]. Among these, DenseFuse incorporates an encoding network composed of convolutional layers, fusion layers, and dense block layers, which significantly enhance its feature extraction capabilities. This design helps mitigate the issue of information loss in the intermediate layers of deep networks. However, maintaining a balance between network performance and the number of parameters remains an open challenge. On the other hand, VAEFuse introduced the use of Variational Autoencoders (VAEs) into the field of image fusion for the first time. Its architecture consists of an image fusion network and an infrared feature compensation network. The encoder processes the infrared and visible light images to generate latent vectors, which are then fused into a unified vector using the product of Gaussian probability densities. The decoder subsequently reconstructs the fused vector into the final fused image. While AE-based methods provide an effective framework for feature extraction and image reconstruction, their complex training processes lead to high computational costs, which remain a significant consideration when deploying these methods.

### 2.3. Vision Transformer

The Transformer architecture, originally introduced by Vaswani et al. for applications in natural language processing (NLP) [16], has since been successfully adapted for computer vision tasks. Its application to infrared and visible image fusion (IVIF) tasks was first pioneered by IFT in 2020 [58]. Building on this foundation, PPTFusion [59] introduced a Transformer-based framework that employs patch Transformers to extract local features and pyramid Transformers to capture global information. Similarly, YDTR [60] proposed a dynamic Transformer capable of simultaneously extracting both local and global features, further advancing the capabilities of Transformers in IVIF tasks. In addition, CDDFuse [19] integrated attention mechanisms into the image fusion framework, utilizing both the long-term and short-term attention capabilities of Transformers to extract shared features across modalities. This approach significantly improved the quality of fused images by effectively aligning information from different sources. However, the high computational demands of most Transformer-based methods have hindered their deployment in mobile and resource-constrained environments compared to their use in larger networks. To address these limitations, Wu et al. proposed LT [61], a lightweight Transformer designed specifically for mobile devices. LT achieves efficiency by combining short- and long-range attention mechanisms with a flattened feedforward network, reducing computational overhead without compromising performance. Compared to traditional Convolutional Neural Networks (CNNs), Transformers offer significant advantages in accelerating model training and inference processes. Nevertheless, their substantial computational resource requirements remain a formidable challenge, particularly for applications requiring real-time processing or deployment on devices with limited hardware capabilities.

## 3. Method

In this section, we first introduce the overall structural framework of the proposed MFRA-Fuse model in Section 3.1. Next, Section 3.2 provides a detailed explanation of the two encoding schemes introduced in this study: Deep Semantic Embedding and Shallow Semantic Embedding. Then, the structure of the fusion module, based on the residual cross-attention mechanism, is thoroughly described in Section 3.3. Finally, the loss function used in our work is presented in Section 3.4.

### 3.1. Framework Overview

**Figure 1:**
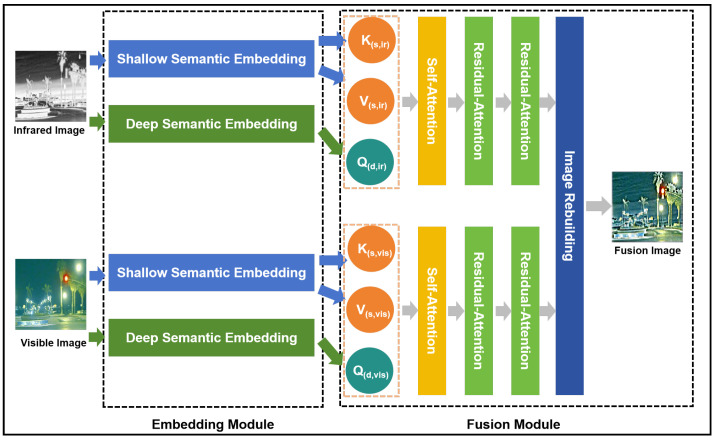
The architecture of our MFRA-Fuse.

As illustrated in Figure 1, the proposed MFRA-Fuse architecture consists of two main components: the embedding module and the fusion module.

*Embedding Module:* Unlike traditional Vision Transformer (ViT) methods, which typically employ a uniform shallow encoding approach for the query, key, and value, this study introduces a deep semantic encoding architecture specifically for encoding the query, while the keys and values are encoded using a shallow encoding architecture. In natural language, each word inherently carries a complete semantic meaning developed over the evolution of human language. By encoding these words and directly computing the similarity scores between the query and key, followed by a linear transformation of the value’s semantic space, tokens can effectively learn accurate contextual semantic relationships. However, in the case of image tokens derived from shallow encodings of image patches, the contextual semantic relationships between tokens are not as strong as in natural language. Therefore, a deeper feature extraction encoding method is necessary to enhance the contextual semantic associations of query tokens in high-dimensional linear spaces.

Nonetheless, deeper semantic encodings tend to deviate from the original image content. If attention computations are performed directly in the deep semantic space, it becomes challenging to preserve the original detailed information of the image. As a solution, we retain the traditional shallow encodings from the original ViT architecture for the key and value components. Ablation experiments in Section 4 validate the effectiveness of the proposed method.

*Fusion Module:* The query (Q), key (K), and value (V) are derived from two distinct encoding strategies: Shallow- and Deep Semantic Embeddings. To better capture the relationships between the encoded tokens generated by these methods, self-attention computations are first performed separately on the Q, K, and V matrices of the infrared and visible images. Following this, two rounds of residual cross-attention computations are applied to integrate information from both the visible and infrared modalities.

Traditional attention modules primarily focus on long-range information, but after multiple iterations of cross-attention in image fusion tasks, the retention of fine-grained details tends to degrade. To address this issue, we propose the residual cross-attention (RCA) mechanism, which introduces a residual connection between the original input value and the output of the attention computation. This residual connection helps preserve the finer details of the images during fusion. The effectiveness of the RCA method is demonstrated through comparative experiments as discussed in Section 4.2.

### 3.2. Embedding Module

**Figure 2:**
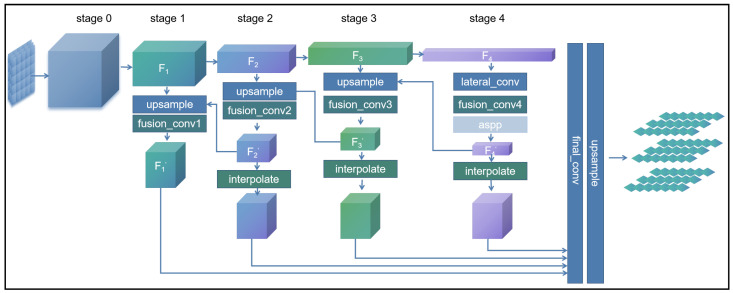
The architecture of Deep Semantic Encoding (DSE).

**Figure 3:**
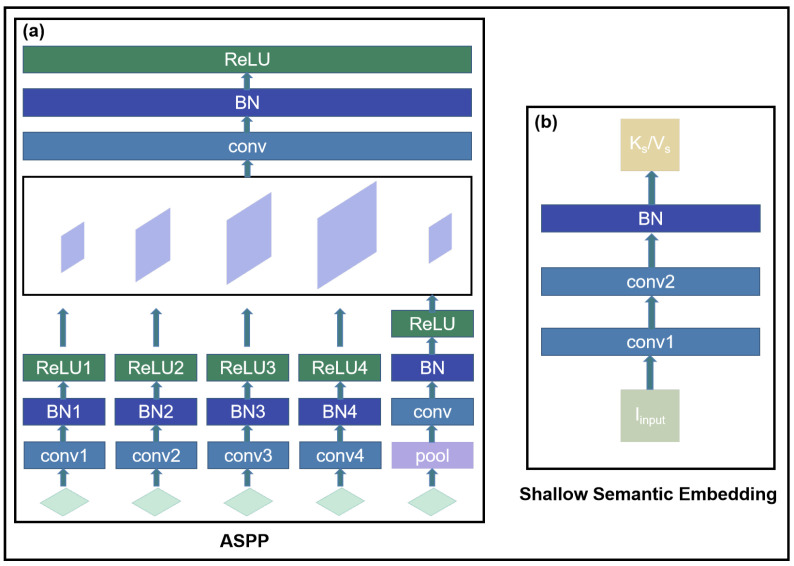
(**a**) The architecture of Atrous Spatial Pyramid Pooling (ASPP); (**b**) the architecture of Shallow Semantic Embedding.

*Deep Semantic Embedding:* The deep semantic encoding architecture is divided into four stages, as shown in Figure 2. The initial encoding scheme for these stages is inspired by the RepVGG design, utilizing the first four layers of the RepVGG-A0 structure. The output features from each stage are denoted as $F_{i}$(i=0,1,2,3,4). To enhance the integration of information across different feature layers, pyramid feature transformation and fusion are applied to $F_{4}$ as described by Equation (1) and illustrated in Figure 3a:

(1) $$F_{4}^{\prime }=ASPP\left(conv\left(F_{4}\right)\right)$$

Subsequently, a recursive computation approach is applied to iteratively process the first three stages in reverse order, as formulated in Equations (2) to (4):

(2) $$F_{3}^{\prime }=conv\left(F_{4}^{\prime }+upsample\left(F_{3}\right)\right)$$

(3) $$F_{2}^{\prime }=conv\left(F_{3}^{\prime }+upsample\left(F_{2}\right)\right)$$

(4) $$F_{1}^{\prime }=conv\left(F_{2}^{\prime }+upsample\left(F_{1}\right)\right)$$

Finally, the cascaded features from the four stages are concatenated and passed through the final encoding process as defined by Equation (5) and denoted as $Q_{d}$:

(5) $$Q_{d}=upsample\left(conv\left(cat\left(interpolate\left(F_{4}^{\prime }\right),interpolate\left(F_{3}^{\prime }\right),interpolate\left(F_{2}^{\prime }\right),F_{1}^{\prime }\right)\right)\right)$$

*Shallow Semantic Embedding:* As mentioned earlier, Shallow Semantic Embedding (SSE) is designed to extract key $\left(K_{s}\right)$ and value $\left(V_{s}\right)$ pairs. During the subsequent residual cross-attention fusion process, the value is connected to the attention computation results via residual connections, helping to better preserve image detail information. To retain more of the original image content, we use only two convolutional layers and a batch normalization layer during the shallow feature extraction stage. The corresponding computation method is defined in Equation (6) and illustrated in Figure 3b:

(6) $$K_{s}/V_{s}=BN\left(conv2\left(conv1\left(I_{input}\right)\right)\right)$$

### 3.3. Fusion Module

**Figure 4:**
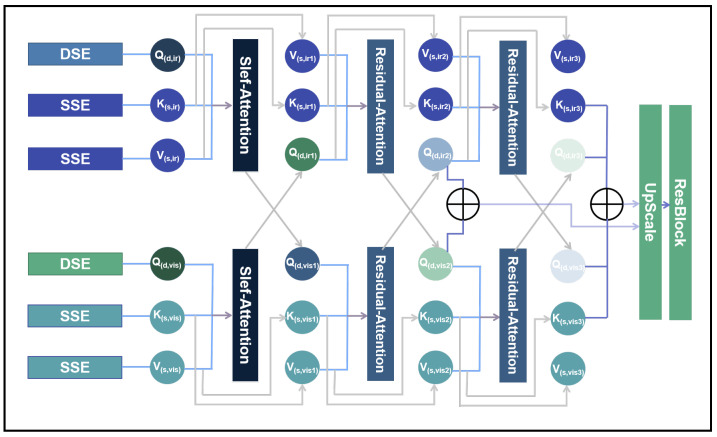
The structure of the residual cross-attention.

In the fusion module, we propose an enhanced approach based on the cross-attention mechanism, introducing the residual cross-attention (RCA) method. As shown in Figure 4, after passing through the deep and shallow feature extraction networks, the infrared images are encoded and represented at each layer as $Q_{\left(d,irn\right)},K_{\left(s,irn\right)},V_{\left(s,irn\right)}$, where (n = 1, 2, 3). Similarly, the visible images are encoded and represented as $Q_{\left(d,visn\right)},K_{\left(s,visn\right)},V_{\left(s,visn\right)}$, where (n = 1, 2, 3). During the residual cross-attention computation, the encoded query, key, and value of the infrared and visible images are first processed separately through the self-attention module. Then, two iterations of the residual cross-attention module are applied.

**Figure 5:**
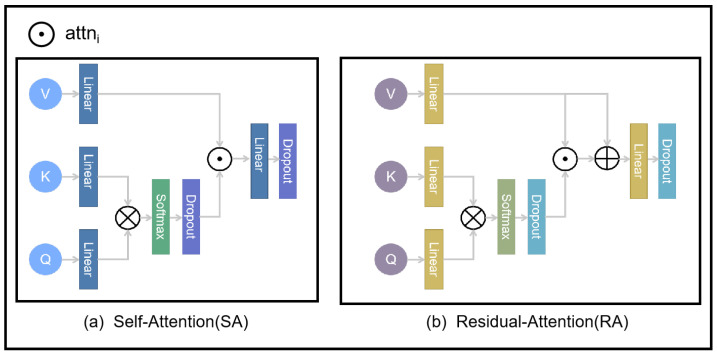
The structure of the SA and RA modules.

First, as shown in Figure 5, the computation methods for both the self-attention and Residual-Attention modules begin with an initial linear transformation of the query, key, and value. This can be expressed by Equations (7) to (9):

(7) $$Q_{\left(d,irn/visn,l\right)}=\mathrm{Linear}Q\left(Q_{\left(d,irn/visn\right)}\right)$$

(8) $$K_{\left(s,irn/visn,l\right)}=\mathrm{Linear}K\left(K_{\left(s,irn/visn\right)}\right)$$

(9) $$V_{\left(s,irn/visn,l\right)}=\mathrm{Linear}V\left(V_{\left(s,irn/visn\right)}\right)$$

The terms $Q_{\left(d,irn/visn,l\right)},K_{\left(s,irn/visn,l\right)},V_{\left(s,irn/visn,l\right)}$ represent the query, key, and value, respectively, following their transformation through linear layers. These projections enable the attention mechanism’s dimensional alignment and feature transformation. The next step is to compute the attention mechanism $attn_{\left(irn/visn\right)}$ (where (n = 1, 2, 3)) using the linearly transformed representations $Q_{\left(d,irn/visn,l\right)}$, $K_{\left(s,irn/visn,l\right)}$, and $V_{\left(s,irn/visn,l\right)}$. This process is formally expressed in Equation (10).

(10) $$attn_{\left(irn/visn\right)}=\mathrm{softmax}\left(\frac{Q_{\left(d,irn/visn,l\right)}K_{\left(s,irn/visn,l\right)}^{T}}{\sqrt{d_{k}}}\right)V_{\left(s,irn/visn,l\right)}$$

The term $d_{k}$ is a scaling factor that mitigates the risk of the softmax function converging to regions with minimal gradients as the dot product values increase. By scaling the dot product, this factor helps maintain more stable gradient magnitudes during the attention mechanism.

First, in the computation of self-attention (SA), no further processing is required for $attn_{\left(irn/visn\right)}$, and its calculation is defined by Equation (11). To better preserve the detailed information of the input images during the fusion process, both attn(irn/visn) and V(s,irn/visn,l) are retained simultaneously in the residual attention fusion process. The structure of the Residual-Attention (RA) module is illustrated in Figure 4, and its corresponding calculation is expressed in Equation (12):

(11) $$SA=attn_{\left(irn/visn\right)}$$

(12) $$RA=V_{\left(s,irn/visn,l\right)}+attn_{\left(irn/visn\right)}$$

In both the self-attention and residual cross-attention mechanisms, the output is further passed through a linear layer and a dropout layer to enhance the stability of the training process. As shown in Figure 4, after two iterations of residual cross-attention fusion, the outputs from both layers are concatenated and fused to jointly reconstruct the fused image. The outputs of the fusion layer are denoted as $FF_{i}$ (where (i = 1, 2)):

(13) $$FF_{1}=\frac{Q_{\left(d,ir2\right)}+Q_{\left(d,vis2\right)}}{2}$$

(14) $$FF_{2}=\frac{Q_{\left(d,ir2\right)}+Q_{\left(d,vis2\right)}+V_{\left(s,ir2\right)}+V_{\left(s,vis2\right)}}{2}$$

The final image reconstruction module consists of a simple MLP. The fused image, denoted as fuse, is computed as shown in Equation (15):

(15) $$fuse=conv(ReLU\left(conv\left(cat\left(FF_{1}+FF_{2}\right)\right)\right))$$

### 3.4. Loss Function

Developing an appropriate loss function is pivotal for improving fusion performance. In this study, the proposed loss function is formulated as Equation (16):

(16) $$L_{total}=\alpha L_{pixel}+\beta L_{texture}+\gamma L_{color}$$

Given the distinct imaging mechanisms of infrared (IR) and visible (VI) images, the loss function proposed in this method ensures that the fusion results effectively preserve sufficient details while emphasizing salient information. Specifically, $L_{pixel}$, $L_{texture}$, and $L_{color}$ represent the pixel loss, texture loss, and color loss, respectively. The hyperparameters α, β, and γ are introduced to balance the contributions of these three loss terms. Based on extensive experiments, Ref. [62] demonstrates that the optimal texture of the fused image can be expressed as the maximum aggregation of the textures from the infrared and visible images. Inspired by Refs. [62,63], the texture details of an image can be effectively represented by the maximum aggregation of its gradients. Accordingly, the texture loss is designed to regulate the gradients of the fused image as formulated in Equation (17):

(17) $$L_{texture}=\frac{1}{HW}||\nabla I_{f}-max\left\{\nabla I_{ir},\nabla I_{vis}\right\}{\left|\right|}_{1}$$

Here, ∇ represents the Sobel operator, which is used to compute the gradient. The notation $\left|\right|\cdot {\left|\right|}_{1}$ denotes the $l_{1}$-norm, and $I_{f}$ refers to the fused image. The variables (H) and (W) represent the height and width of the image, respectively, while the operation (max) indicates the element-wise maximum selection.

For pixel-wise loss, this paper proposes an infrared and visible image fusion loss function based on gradient-adaptive weighting. Specifically, the gradient information of the two input images is first computed using the Sobel operator. The larger gradient value at each pixel location is selected by comparison between the two images. To facilitate unified computation and obtain smoother weighting factors, the selected gradient values are transformed using a softmax function, which serves as the adaptive loss weight:

(18) $$grad\_weiht=softmax\{max\left\{\nabla I_{ir},\nabla I_{vis}\right\}\}$$

where ∇ represents the Sobel operator employed to compute the gradient. This approach preserves pixels with rapid gradient changes in the texture-detailed regions of the images, while in regions with smooth color transitions, the fused image is encouraged to approximate the visible image. This is because visible images typically capture richer color information, which is desirable for the final fused result. The notation ${\left||\cdot |\right|}_{1}$ represents the Smooth l_1 norm:

(19) $$I_{target}=grad\_weiht*max\left\{I_{ir},I_{vis}\right\}+\left(1-grad\_weiht\right)*I_{vis}$$

(20) $$L_{pixel}=\left|\right|I_{f}-I_{target}{\left|\right|}_{1}$$

The color loss is divided into two components.

(21) $$L_{color}=L_{pixel\_color}+L_{color\_consistency}$$

Since thermal infrared imaging systems output grayscale images without color information, the first component focuses on ensuring that the fused image in the RGB color space closely resembles the visible image in terms of visual perception to the human eye.

(22) $$L_{pixel\_color}=\frac{1}{HW}\sum \underset{0}{H}\sum \underset{0}{W}{\left(I_{f}-I_{vis}\right)}^{2}$$

To ensure that the fused image achieves a more harmonious appearance in the RGB color space, the images are first separated into their RGB components. The mean of the red channel from both the fused and visible light images is calculated to serve as a weighting factor for the Euclidean distance calculation in the color space for the red and blue channels:

(23) $$R_{mean}=\left(R_{f}+R_{vis}\right)/2$$

(24) $$R=R_{vis}-R_{f}$$

(25) $$G=G_{vis}-G_{f}$$

(26) $$B=B_{vis}-B_{f}$$

Meanwhile, the weight for the green channel is fixed at four. This weighting strategy takes into account the varying sensitivity of the human visual system to different color channels, with green typically being perceived as the most sensitive and therefore given greater weight:

(27) $$color\_distance=\sqrt{(2+\left(R_{mean}/256\right)}*R^{2}+4*G^{2}+\sqrt{(2-\left(R_{mean}/256\right)}*B^{2}$$

Finally, the Euclidean distance is transformed through an exponential function to obtain a color space similarity score:

(28) $$L_{color\_consistency}=1-e^{\left(-color\_distance/255\right)}$$

## 4. Experiments and Results

In this section, we first introduce the datasets and provide a detailed description of the implementation settings. Next, we outline the comparative experimental methodologies and the objective evaluation metrics employed in this study. Following that, we present and analyze the experimental results, accompanied by a comprehensive discussion. Furthermore, ablation studies are conducted to investigate the contributions of individual components in greater detail.

### 4.1. Datasets and Implementation Settings

In this study, we use the publicly available RoadScene and M3FD datasets, selecting 219 pairs of infrared and visible (IV) images from each. The RoadScene dataset covers a diverse array of scenarios, including roadways, pedestrians, and other common elements, while the M3FD dataset offers source images captured under challenging conditions, such as intense glare, low-light environments, and complex weather phenomena including rain and fog. These two datasets are key resources for image fusion in real-world scenarios. Additionally, we employ the Harvard dataset, a medical imaging repository provided by Harvard Medical School, which contains both normal and pathological brain images across multiple modalities, including MRI, PET, and SPECT. These images are randomly cropped into sample patches of size 128×128 for training. To train the proposed MFRA-Fuse model, we utilize the AdamW optimizer with an initial learning rate of $2\times 10^{-3}$ and a weight decay of $1\times 10^{-2}$. All input images are randomly cropped to a size of 128×128. The experiments are conducted using an NVIDIA GeForce RTX 3090 GPU, which is designed and manufactured by NVIDIA Corporation and the framework is implemented in PyTorch.

### 4.2. Comparative Methods and Objective Evaluation Metrics

*Comparative Methods*: To evaluate the effectiveness and superiority of the proposed method, comparisons are conducted with six state-of-the-art approaches, including two variants of DenseFusion [64] (one using the addition loss function and the other employing the L1-norm loss function), LRRNet [65], RFN-Nest [66], SwinFusion [18], and U2Fusion [9]. The source codes for all the compared methods are publicly available, and their parameters are configured following the recommendations provided in their respective original papers to ensure a fair and accurate comparison.

*Objective Evaluation Metrics*: In this study, seven objective evaluation metrics are selected to comprehensively assess the performance of image fusion methods, each capturing different aspects of the fused image’s quality:

(29) $$EN=-\sum_{n=1}^{N}P_{f}\ln P_{f}$$

Information-based metrics include EN (entropy). Following Equation (29), $P=p_{1},p_{2},...,p_{n}$, which denotes the grayscale intensity distribution of an image, where N is the total number of discrete grayscale levels and each $p_{i}$ represents the corresponding probability (or frequency) of the i-th level, which quantifies the amount of information contained in the fused image,

(30) $$MI_{F,X}\left(f;x\right)=\sum_{f,x}P_{F,X}\left(f,x\right)\ln\frac{P_{F,X}\left(f,x\right)}{P_{F}\left(f\right)P_{X}\left(x\right)}$$

and MI (Mutual Information). In this context,$P_{F}\left(f\right)$ and $P_{X}\left(x\right)$ represent the edge distribution histograms of the fused image F and the source image X, respectively. $P_{F,X}$(f,x) denotes the joint distribution histogram of the fused image F and the source image X which measures the amount of information transferred from the source images to the fused image:

(31) $$AG=\frac{1}{MN}\sum_{i=1}^{M}\sum_{j=1}^{N}\sqrt{\frac{{\left(F(i+1,j)-F(i,j)\right)}^{2}+{\left(F(i,j+1)-F(i,j)\right)}^{2}}{2}}$$

Feature-based metrics include AG (Average Gradient), which reflects the richness of texture and detail information by measuring the gradient information of the fused image,

(32) $$SF=\sqrt{RF^{2}+CF^{2}}$$

(33) $$RF=\sqrt{\sum_{i=1}^{M}\sum_{j=1}^{N}{\left(F(i,j)-F(i,j-1)\right)}^{2}}$$

(34) $$CF=\sqrt{\sum_{i=1}^{M}\sum_{j=1}^{N}{\left(F\left(i,j\right)-F\left(i-1,j\right)\right)}^{2}}$$

SF (Spatial Frequency), RF, which represents the row frequency, and CF, which represents the column frequency and which evaluates the preservation of texture and detail information from the source images in the fused image:

(35) $$SD=\sqrt{\sum_{i=1}^{M}\sum_{j=1}^{N}{\left(F(i,j)-\mu \right)}^{2}}$$

where SD (Standard Deviation) characterizes the contrast distribution of the fused image. In Equation (35), μ represents the mean gray level of the fused image. Given that the human visual system is inherently more sensitive to regions with higher contrast, areas with greater contrast in the fusion process are perceived more favorably. Therefore, higher contrast generally leads to better fusion results:

(36) CC=ωrofo+ωrufu

(37) $$r_{xf}=\frac{\sum_{i=1}^{M}\sum_{j=1}^{N}\left(x_{i,j}-\mu_{x}\right)\left(f_{i,j}-\mu_{f}\right)}{\sqrt{\sum_{i=1}^{M}\sum_{j=1}^{N}{\left(x_{i,j}-\mu_{x}\right)}^{2}\sum_{i=1}^{M}\sum_{j=1}^{N}{\left(f_{i,j}-\mu_{f}\right)}^{2}}}$$

Additionally, the correlation-based metric CC (Correlation Coefficient) is utilized to evaluate the linear relationship between the fused image and the source images. In Equation (36), $\omega_{o}$ and $\omega_{u}$ are typically set to 0.5. In Equation (37), $\mu_{x}$ and $\mu_{f}$ represent the mean values of the source image *x* and the fused image *f*, respectively:

(38) $$VIF=\frac{VIF_{A,F}+VIF_{B,F}}{2}$$

(39) $$VIF_{X,F}=\frac{\sum_{j\in subbands}I\left(C^{S,i};F^{S,i}|R^{S,i}\right)}{\sum_{j\in subbands}I\left(C^{S,i};X^{S,i}|R^{S,i}\right)}$$

And, the perception-based metric VIF (Visual Information Fidelity) objectively evaluates the fused image’s quality by reflecting the subjective visual perception of human observers. In Equation (39), $I(C^{S,i};X^{S,i}|R^{S,i})$ and $I(C^{S,i};F^{S,i}|R^{S,i})$ represent the amount of information obtained by the human brain from the source images and the fused results, respectively. $C^{S,i}$ is defined as the vector of all blocks in a specific subband. $X^{S,i}$ and $F^{S,i}$ denote the *S*-th elements derived from $X^{i}$ (the output signal of the HSV model with the *i*-th subband of the source image as input) and $F^{i}$ (the output signal of the HSV model with the *i*-th subband of the fused image as input), respectively. Moreover, $R^{S,i}$ represents the model parameters of the *i*-th subband. A higher VIF value indicates that the generated image is more consistent with the human visual perception system.

(40) $$SSIM\left(I_{F},I_{*}\right)=\sum_{I_{r},I_{*}}\frac{2\mu_{I_{r}}\mu_{I_{*}}+C_{1}}{\mu_{I_{r}}^{2}+\mu_{I_{*}}^{2}+C_{1}}\times \frac{2\sigma_{I_{r}}\sigma_{I_{*}}+C_{2}}{\sigma_{I_{r}}^{2}+\sigma_{I_{*}}^{2}+C_{2}}\times \frac{\sigma_{I_{r}I_{*}}+C_{3}}{\sigma_{I_{r}}\sigma_{I_{*}}+C_{3}}$$

Finally, the Structural Similarity Index Measure (SSIM) is employed to assess structural differences. This metric evaluates three key aspects of information: luminance, structure, and contrast, as defined in Equation (40). In this context, $I_{*}$ represents the source image ($I_{A}$ or $I_{B}$), while μ and σ denote the mean and standard deviation, respectively. The constants $C_{1}$, $C_{2}$, and $C_{3}$ are introduced to avoid division by zero. A higher structural similarity score indicates reduced information loss during the fusion process, thereby reflecting better performance of the fused image.

### 4.3. Results and Discussion

**Figure 6:**
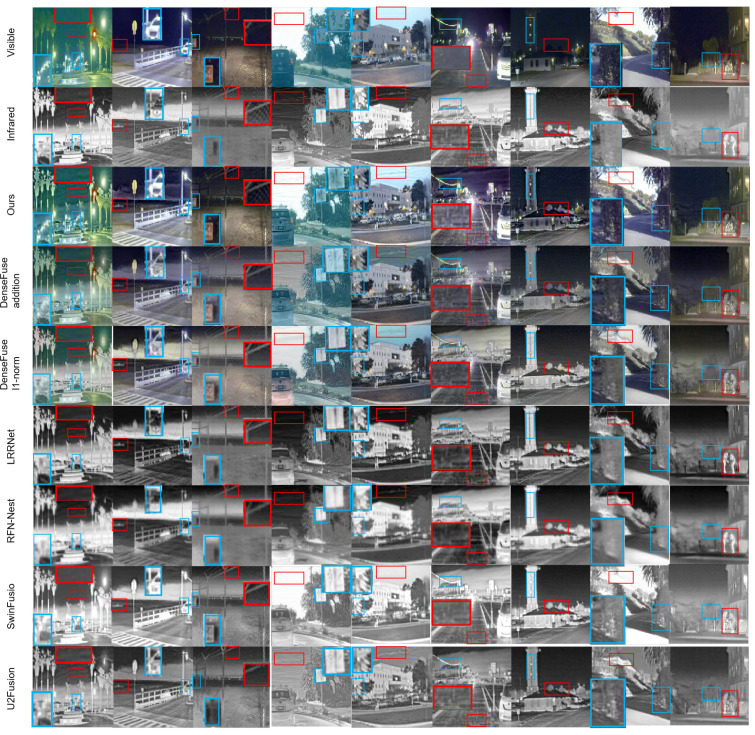
The results of fusing nine pairs of visible and infrared images from the RoadScene dataset.The red box indicates clear textures in the infrared image, and the blue box represents clear textures in the visible light image.

As illustrated in Figure 6, the fusion results for nine pairs of visible and infrared images from the RoadScene dataset are presented, with each image emphasizing two specific details. Although all seven methods demonstrate varying degrees of effectiveness in image fusion, six of them reveal certain limitations or deficiencies in preserving or enhancing these finer details during the fusion process.

It can be observed that, in terms of texture details, both LRRNet and RFN-Nest exhibit relatively inferior performance, with blurred image edges. This is particularly evident in the comparisons within the red box in the third column and the blue box in the fourth column (from left to right). In the third column, only the proposed method (Ours) and SwinFusion manage to preserve the intertwined grid-like texture. In the fourth column, the road sign information is most comprehensively retained by the proposed method (Ours), while DenseFusion (with the addition of a loss function) and SwinFusion also manage to preserve some contours.

Regarding color style, although DenseFusion generates color images, inconsistencies in color representation are noticeable compared to the original visible-light images. This is especially evident in the first column on the left, where the color of the leaves deviates significantly from the original. This further highlights the effectiveness of the proposed color loss function, which enables the output images to better preserve a color style closer to the true scene. However, there is still room for improvement in both the proposed method (Ours) and SwinFusion in certain specific details. For instance, in the eighth image from the left, the building is only partially represented by its edge contours. This limitation arises because the attention mechanism prioritizes global information, while the proposed method emphasizes maintaining consistency between the color style of the fused image and the real-world scene. As a result, texture detail recovery in areas of high exposure remains insufficient.

**Figure 7:**
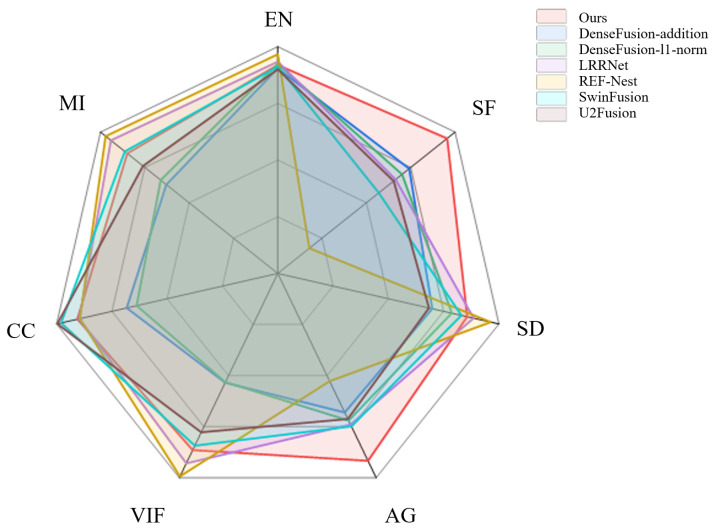
Performance comparison of our method with existing image fusion approaches.

**Figure 8:**
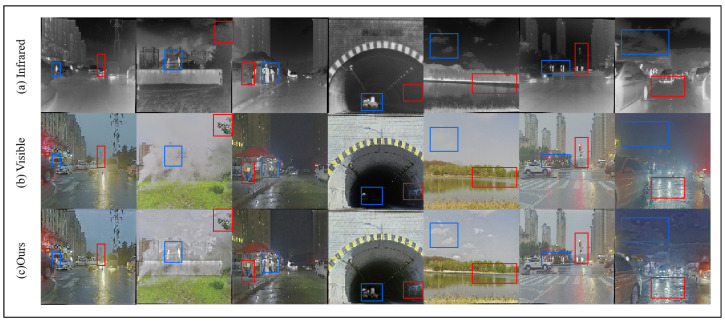
The results of image fusion on the M3FD dataset.

Based on the objective metrics evaluated across the seven methods as shown in Table 1 and Figure 7, our proposed method achieves the best performance in both SF and AG. For the remaining metrics, it consistently ranks within the top three. Furthermore, unlike other methods, our approach does not exhibit any significant weaknesses in specific metrics. For example, DenseFusion performs notably worse in CC, while RFN-Net shows poor performance in SF. These findings further emphasize the balanced and robust performance of our method across various evaluation criteria. Additionally, to validate the generalization capability of the proposed method, we conducted experiments on the M3FD dataset. The fusion results on the M3FD dataset are shown in Figure 8.

**Figure 9:**
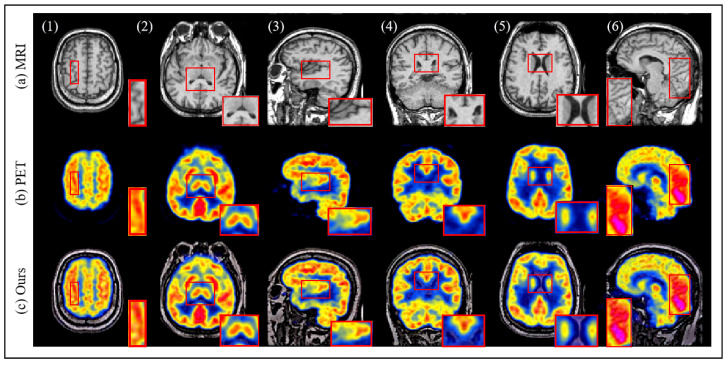
The results of image fusion on the Harvard dataset.The red box displays the texture details of the PET image, MRI image and the fused image.

To further evaluate the robustness of the proposed method, we utilize the Harvard dataset for testing. The fusion results are depicted in Figure 9. Based on the data provided in [9], we compare the proposed method with six other medical image fusion techniques. As shown in Table 2, the proposed method ranks within the top three across all four evaluated metrics, with SSIM achieving the best performance among the six methods. Additionally, ablation studies conducted on both datasets not only confirm the effectiveness of the proposed method but also underscore its strong generalization capability.

### 4.4. Ablation Studies

In this section, we conduct three ablation experiments to evaluate the effectiveness of Shallow and Deep Semantic Embeddings, the contribution of the RCA structure, and the improvements introduced by the deep encoding architecture. Furthermore, we analyze specific configurations, investigate the impact of the hyperparameters α, β, and γ, discuss the rationale behind the selection of various optimization algorithms, and provide a comparative evaluation of different normalization functions.

*Shallow and Deep Semantic Embedding:* To evaluate the effectiveness of applying Deep Semantic Embedding and Shallow Semantic Embedding to the encoding of Q, K, and V, we conduct experiments in which the Q, K, and V of the RCA module are encoded using the same shallow embedding. Additionally, experiments are performed where Q is encoded using the Deep Semantic Embedding (DSE) module, while K and V are encoded using the Shallow Semantic Embedding (SSE) module.

**Figure 10:**
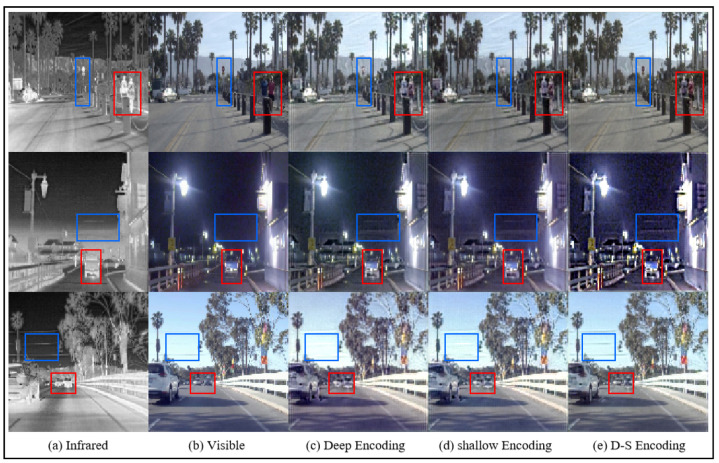
The results of deep embedding, shallow embedding, and the proposed deep–shallow embedding image fusion on the M3FD dataset. The blue box indicates clear textures in the infrared image, and the red box represents clear textures in the visible light image.

Validation on the RoadSense and M3FD datasets as shown in Table 3, reveals that the DSE + RCA method achieves significant improvements in the SF, SD, and VIF metrics, with a slight decrease in AG, while other metrics remain generally stable. The overall comparison of metrics demonstrates that incorporating Deep Semantic Embedding enhances the effectiveness of image information fusion. Furthermore, as illustrated in Figure 10, the fusion results of the three methods, namely deep embedding, shallow embedding, and the proposed deep–shallow embedding, are visually compared. It is evident that details such as the person, the blue car, and the fine electrical wires in the sky are most clearly preserved with the deep–shallow embedding approach.

**Figure 11:**
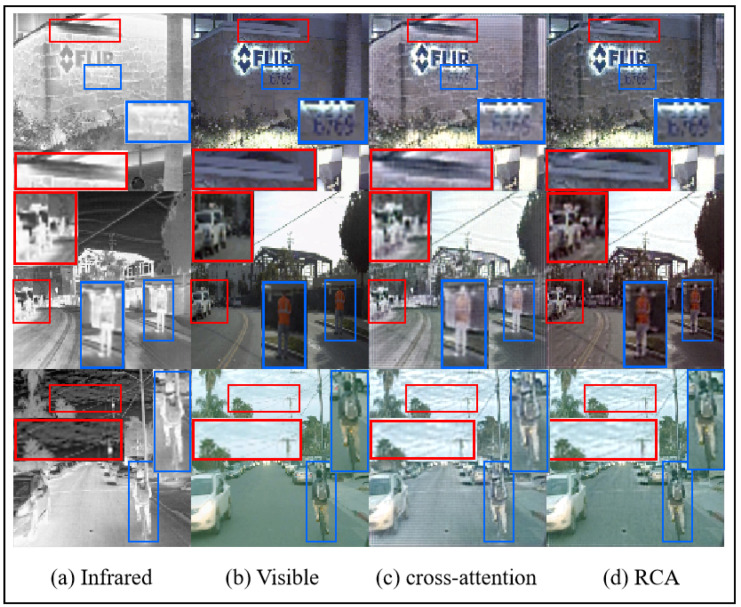
The results of RCA and cross-attention image fusion on the M3FD dataset.The red box indicates clear textures in the infrared image, and the blue box represents clear textures in the visible light image.

*RCA Module:* Attention mechanisms often prioritize global information, which can lead to the transformation of original input features and the loss of some details during long-range computations. To better preserve the original fine-grained features in fused images, we propose the residual cross-attention (RCA) mechanism. A comparison of the metrics for images fused using the RCA module versus those fused with a standard cross-attention mechanism, as shown in Table 4, demonstrates that the RCA module effectively enhances the SD, AG, VIF, and MI metrics across both datasets, while the remaining metrics remain largely unaffected. Furthermore, Figure 11 provides a visual comparison of fusion results, highlighting the RCA method’s superiority. With RCA, both fine details, such as the numbers and the car in the corner, and primary texture information, such as the person and the clouds in the sky, are more clearly preserved in comparison to the standard cross-attention approach.

*Deep Semantic Embedding Architecture:* In this paper, we propose an enhanced Deep Semantic Embedding method for image fusion tasks by improving the RepVGG model with pyramid semantic extraction and cascade fusion feature encoding structures. To evaluate the effectiveness of this approach, we conduct comparisons across three fusion methods on two datasets: (1) using only the RCA structure, (2) using the RepVGG + RCA structure, and (3) using the DSE + RCA structure. The results, presented in Table 5, show that while employing RepVGG as the deep semantic encoder yields only marginal improvements in image fusion metrics compared to using the RCA model alone, using DSE as the deep semantic encoder achieves significant improvements across multiple metrics. These findings demonstrate that the proposed method effectively enhances the extraction of query features for attention mechanism calculations.

**Figure 12:**
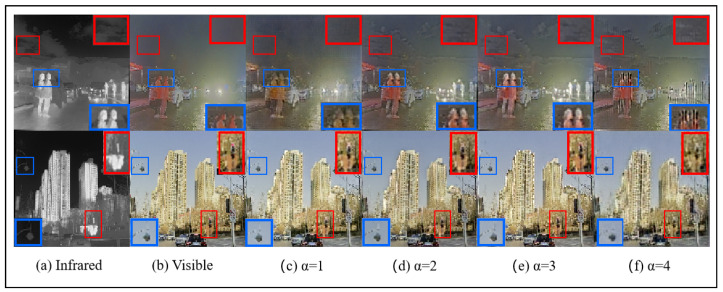
The results of different α set fusion image on the M3FD dataset. The blue box indicates clear textures in the infrared image, and the red box represents clear textures in the visible light image.

*Hyperparameter α:* As discussed in Section 3.3, the hyperparameter α is used to regulate pixel loss. In our experiments, α is set to 1, 2, 3, and 4. The results, summarized in Table 6, show that the overall performance metrics are optimal when α = 3. Additionally, the visual comparisons in Figure 12 highlight that texture details are best preserved when α = 3. When α is set to smaller values, image details—such as the contours of human figures, the background sky, and cloud textures—appear noticeably blurred. Conversely, when α = 4, an over-sharpening effect becomes apparent.

**Figure 13:**
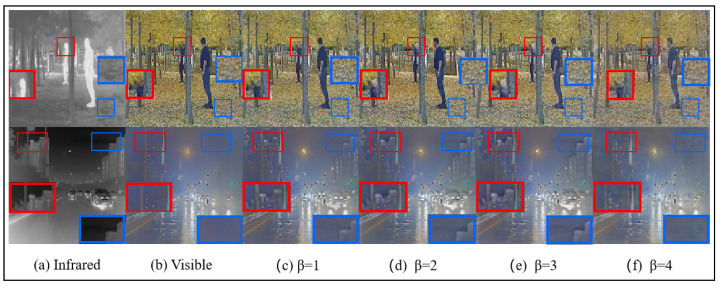
The results of different β set fusion image on the M3FD dataset.The blue box indicates clear textures in the infrared image, and the red box represents clear textures in the visible light image.

*Hyperparameter β:*β is a crucial hyperparameter used to regulate texture loss in image fusion. To evaluate its effect, we conduct comparative experiments with β set to 1, 2, 3, and 4. The results of the image fusion metrics under varying β values are summarized in Table 7. The overall performance is optimal when β is set to either 2 or 3. However, as illustrated in Figure 13, when β=3, details such as the windows of buildings and the contours of human figures are more distinctly preserved.

**Figure 14:**
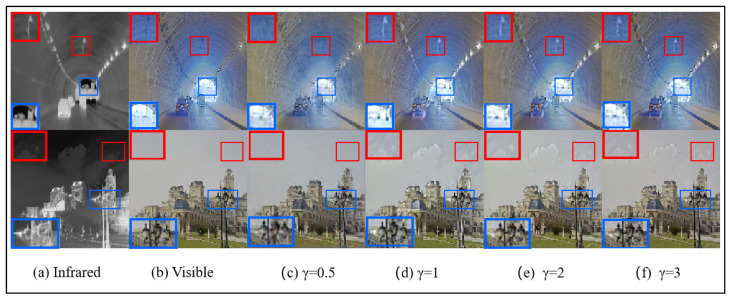
The results of different γ set fusion image on the M3FD dataset.The blue box indicates clear textures in the infrared image, and the red box represents clear textures in the visible light image.

*Hyperparameter γ:* As discussed in Section 3.3, γ is a key hyperparameter that controls the color loss in image fusion. To assess its impact, we conduct a series of comparative experiments with γ set to 0.5, 1, 2, and 3. The results, summarized in Table 8, indicate that the overall performance metrics are optimized when γ=1. Additionally, a visual comparison provided in Figure 14 illustrates that when γ=1, finer details—such as the buildings at the end of the tunnel and the intricate shapes of the streetlights—are better preserved.

**Figure 15:**
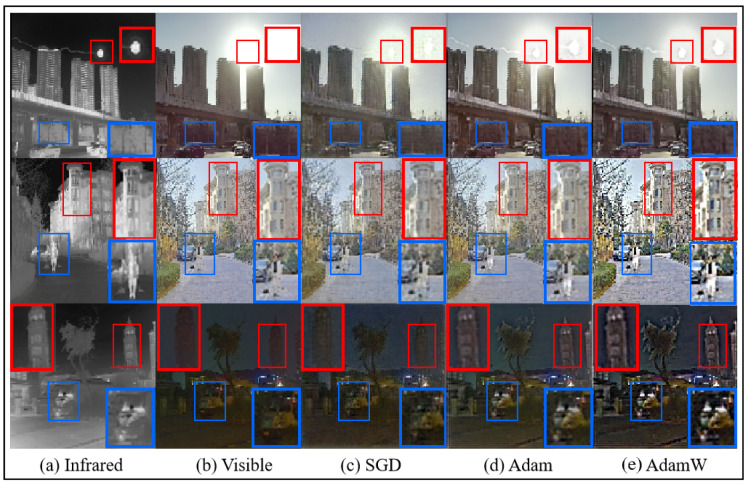
The results of different optimizers set fusion image on the M3FD dataset.The blue box indicates clear textures in the infrared image, and the red box represents clear textures in the visible light image.

*Optimizer*: To enhance the training performance of the proposed model, we compare three widely used optimizers in deep learning: SGD, Adam, and AdamW. The test results, shown in Figure 15, indicate that the AdamW optimizer achieves superior performance compared to the others. Specifically, AdamW demonstrates optimal results in preserving details such as human subjects, architectural elements, high-exposure sunlight, and shadow textures. Based on these findings, this study adopts AdamW as the optimizer for model training.

**Figure 16:**
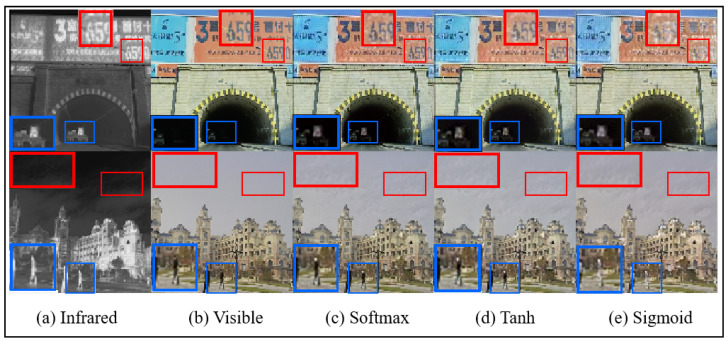
The results of different normalization function set fusion images on the M3FD dataset.The blue box indicates clear textures in the infrared image, and the red box represents clear textures in the visible light image.

*Normalization Function*: In Section 3.3, the softmax function is selected as the normalization method for computing grad_weight in the texture loss function. To assess the impact of different normalization functions on image fusion performance, we conduct comparative experiments using three alternatives: sigmoid, softmax, and tanh. As shown in Table 9, the overall performance of all three normalization functions is relatively comparable in terms of quantitative image fusion metrics. However, as illustrated in Figure 16, the softmax function produces superior visual fusion results, particularly in preserving fine details such as human contours, text on billboards, and vehicles in darker areas such as tunnels. Based on these findings, the softmax function is adopted as the normalization method for computing grad_weight in this study.

*Computational Efficiency*: Based on the data presented in [7], the average computation times of eight different fusion methods on the RoadScene dataset are as follows: DATFuse: 0.1248 s; DenseFuse: 0.1082 s; FusionGAN: 0.1401 s; RFN-Nest: 0.1515 s; SwinFusion: 0.9637 s; U2Fusion: 0.0682 s; ATFFuse: 0.1081 s; and the proposed method: 0.135 s. The computational speed of our method is moderate compared to other image fusion methods. This is primarily attributed to the multiple feature extraction and data transformation steps involved, which introduce additional computational overhead. Nonetheless, this slight increase in computation time is a reasonable trade-off, as the proposed method achieves significantly improved image fusion performance as demonstrated in the earlier sections.

## 5. Conclusions

This paper addresses the limitations of existing encoding methods in Transformer architectures when applied to the image domain, where image tokens often lack the precise and meaningful semantics that natural language tokens possess. Moreover, preserving the fine details of the original input images remains a significant challenge in image fusion tasks. To tackle these issues, we propose an innovative deep–shallow semantic encoding structure. Building on this foundation, we introduce a multi-stage cascade fusion strategy that leverages deep semantic encoding. In addition, we are the first to propose a residual cross-attention module, designed to retain more of the original image details during long-range computations. To further enhance adaptability to diverse image scenarios, we devise a novel fusion function based on image gradients and color style control.

Comprehensive ablation experiments were conducted on the RoadScene and M3FD datasets, comparing our method with six state-of-the-art techniques. The results demonstrate that the proposed approach achieves superior overall performance in terms of both qualitative and quantitative evaluations. Looking ahead, future work will focus on enhancing the fusion efficiency and adaptability of the proposed method. Specifically, we aim to design a structure capable of automatically selecting the depth of feature encoding layers, achieving an optimal balance between shallow and deep features. Additionally, we plan to streamline the image reconstruction process to reduce computational overhead while further improving the method’s adaptability across various image fusion scenarios.

## Figures and Tables

**Table 1:** Performance comparison of our method with existing image fusion approaches.

Method	Ours	Densefusion -Addition	Densefusion -l1norm	LRRNet	RFN-Net	Swinfusion	U2fusion
EN	7.3539	7.2388	7.3953	7.4726	7.7313	7.2977	7.2021
SF	10.8656	10.2246	10.104	9.9933	8.5346	9.7095	9.9612
SD	54.245	42.424	49.0352	56.424	62.2731	52.2909	41.2881
AG	11.0065	8.1662	8.6418	8.8726	6.3249	8.9826	8.547
VIF	0.7295	0.0694	0.0647	0.8587	0.987	0.6854	0.5552
CC	0.4956	0.1276	0.053	0.4802	0.477	0.617	0.6458
MI	4.5185	3.0844	3.2904	5.1153	5.3079	4.6078	3.9394

**Table 2:** Four Metrics on Medical Image Fusion on the Harvard Dataset.

Method	SCD	CC	SSIM	PSNR
RPCNN	1.429	0.785	1.9865	61.213
CNN	1.272	0.798	1.9885	62.137
PAPCNN	1.289	0.784	1.9872	61.565
NSCT	0.969	0.769	1.9875	61.695
FusionDN	0.742	0.805	1.9769	59.178
U2Fusion	1.312	0.834	1.9921	63.458
Ours	1.3084	0.812	1.9930	62.6568

**Table 3:** Comparison of DSE + RCA and RCA under different settings.

Method	DSE + RCA (RS)	RCA (RS)	DSE + RCA (M3)	RCA (M3)
EN	7.3539	7.3532	6.7297	6.7279
SF	10.8656	10.8112	9.6882	9.6595
SD	54.245	54.0624	34.8675	34.6817
AG	11.0065	11.1028	8.2538	8.3246
VIF	0.7546	0.7295	0.8604	0.8569
CC	0.4956	0.4905	0.4272	0.4256
MI	4.5185	4.6491	4.5065	4.6640

**Table 4:** Comparison of DSE + RCA and DSE + cross under different settings.

Method	DSE + RCA (RS)	DSE + Cross (RS)	DSE + RCA (M3)	DSE + Cross
EN	7.3539	7.3655	6.7297	6.7171
SF	10.8656	10.9616	9.6882	9.6388
SD	54.245	51.6063	34.8675	34.3678
AG	11.0065	10.8373	8.2538	8.0898
VIF	0.7546	0.6837	0.8604	0.8438
CC	0.4956	0.5373	0.4272	0.4268
MI	4.5185	4.3507	4.5065	4.3463

**Table 5:** Comparison of RCA, RepVgg + RCA, and DSE + RCA under different settings.

Method	RCA (RS)	RepVgg + RCA (RS)	DSE + RCA (RS)	RCA (M3)	RepVgg + RCA (M3)	DSE + RCA (M3)
EN	7.3532	7.356	7.3539	6.7279	6.7184	6.7297
SF	10.8112	10.7838	10.8656	9.6595	9.6199	9.6882
SD	54.0624	54.065	54.245	34.6817	34.6885	34.8675
AG	11.1028	10.8822	11.0065	8.3246	8.2282	8.2538
VIF	0.7295	0.726	0.7546	0.8569	0.8574	0.8604
CC	0.4905	0.4957	0.4956	0.4256	0.4269	0.4272
MI	4.6491	4.4679	4.5185	4.6640	4.5462	4.5065

**Table 6:** Comparison of α under different settings.

Method	α = 1	α = 2	α = 3	α = 4
EN	6.6723	6.7249	6.7322	6.7356
SF	9.4731	9.7064	9.8673	9.643
SD	34.0763	34.8156	34.7173	34.675
AG	7.9214	8.2547	8.4242	8.3083
VIF	0.9098	0.8588	0.8752	0.8340
CC	0.4138	0.4249	0.3525	0.3478
MI	4.7123	4.5494	4.5934	4.5836
Qabf	0.021	0.0204	0.0245	0.0208
SSIM	1.6402	1.6404	1.635	1.6257

**Table 7:** Comparison of β under different settings.

Method	β = 1	β = 2	β = 3	β = 4
EN	6.6749	6.7249	6.7319	6.6924
SF	9.6396	9.7064	9.7156	9.6678
SD	34.043	34.8156	34.834	34.743
AG	7.9095	8.2547	8.3085	7.9995
VIF	0.9166	0.8588	0.852	0.8235
CC	0.4097	0.4249	0.4278	0.4147
MI	4.5191	4.5494	4.5977	4.5486
Qabf	0.0209	0.0204	0.0202	0.0205
SSIM	1.6395	1.6404	1.6418	1.6324

**Table 8:** Comparison of β under different settings.

Method	γ = 0.5	γ = 1	γ = 2	γ = 3
EN	6.7167	6.7709	6.7249	6.692
SF	9.7542	9.9105	9.7064	9.5391
SD	34.6367	35.1361	34.8156	34.4631
AG	8.391	8.5175	8.2547	8.0786
VIF	0.8017	0.8024	0.8588	0.8948
CC	0.4231	0.4472	0.4249	0.4149
MI	4.624	4.821	4.5494	4.6926
Qabf	0.021	0.0198	0.0204	0.0207
SSIM	1.6401	1.6451	1.6404	1.6453

**Table 9:** Comparison of normalization function under different settings.

Method	Sigmoid	Tanh	Softmax
EN	6.7249	6.7229	6.7232
SF	9.7064	9.7	9.6703
SD	34.8156	34.8385	34.8224
AG	8.2547	8.3	8.3137
VIF	0.8588	0.8622	0.8787
CC	0.4249	0.4225	0.4186
MI	4.5494	4.5704	4.6938
Qabf	0.0204	0.0204	0.0203
SSIM	1.6404	1.6397	1.6398

## Data Availability

Publicly available datasets were analyzed in this study.
